# Facile Fabrication of Natural Polyelectrolyte-Nanoclay Composites: Halloysite Nanotubes, Nucleotides and DNA Study

**DOI:** 10.3390/molecules25153557

**Published:** 2020-08-04

**Authors:** Svetlana Batasheva, Marina Kryuchkova, Ramil Fakhrullin, Giuseppe Cavallaro, Giuseppe Lazzara, Farida Akhatova, Läysän Nigamatzyanova, Vladimir Evtugyn, Elvira Rozhina, Rawil Fakhrullin

**Affiliations:** 1 Institute of Fundamental Medicine and Biology, Kazan Federal University, Kreml uramı 18, Kazan, Republic of Tatarstan 420008, Russia; maricshka80@gmail.com (M.K.); ramilfahrullin@gmail.com (R.F.); akhatovaf@gmail.com (F.A.); lyaysan.nigamatzyanova@gmail.com (L.N.); VGEvtjugin@kpfu.ru (V.E.); rozhinaelvira@gmail.com (E.R.); 2 Dipartimento di Fisica e Chimica, Università di Palermo, Viale delle Scienze pad 17, 90128 Palermo, Italy; giuseppe.cavallaro@unipa.it (G.C.); giuseppe.lazzara@unipa.it (G.L.)

**Keywords:** clay/polymer composites, halloysite nanotubes, nanoclay, DNA, nucleotide, nanoclay self-assembly

## Abstract

Complexation of biopolymers with halloysite nanotubes (HNTs) can greatly affect their applicability as materials building blocks. Here we have performed a systematic investigation of fabrication of halloysite nanotubes complexes with nucleotides and genomic DNA. The binding of DNA and various nucleotide species (polyAU, UMP Na_2_, ADP Na_3_, dATP Na, AMP, uridine, ATP Mg) by halloysite nanotubes was tested using UV-spectroscopy. The study revealed that binding of different nucleotides to the nanoclay varied but was low both in the presence and absence of MgCl_2_, while MgCl_2_ facilitated significantly the binding of longer molecules such as DNA and polyAU. Modification of the nanotubes with DNA and nucleotide species was further confirmed by measurements of ζ-potentials. DNA-Mg-modified nanotubes were characterized using transmission electron (TEM), atomic force (AFM) and hyperspectral microscopies. Thermogravimetric analysis corroborated the sorption of DNA by the nanotubes, and the presence of DNA on the nanotube surface was indicated by changes in the surface adhesion force measured by AFM. DNA bound by halloysite in the presence of MgCl_2_ could be partially released after addition of phosphate buffered saline. DNA binding and release from halloysite nanotubes was tested in the range of MgCl_2_ concentrations (10–100 mM). Even low MgCl_2_ concentrations significantly increased DNA sorption to halloysite, and the binding was leveled off at about 60 mM. DNA-Mg-modified halloysite nanotubes were used for obtaining a regular pattern on a glass surface by evaporation induced self-assembly process. The obtained spiral-like pattern was highly stable and resisted dissolution after water addition. Our results encompassing modification of non-toxic clay nanotubes with a natural polyanion DNA will find applications for construction of gene delivery vehicles and for halloysite self-assembly on various surfaces (such as skin or hair).

## 1. Introduction

Clay-based composite materials have found numerous applications in recent years, opening new opportunities in materials science and biomedicine [1] Tailoring various biopolymers to clay particles allows for controllable self-assembly on planar and three-dimensional surfaces and fabrication of porous clay-doped polymer composites, which can be utilised in tissue engineering [2], artificial cell shellization [3] and hair surface engineering [4]. The invention of gene therapy is one of the greatest achievements of modern medicine because of its potential of overcoming cancer and serious inherited illnesses (such as neurodegenerative diseases or blood and immune disorders) that once were believed to be incurable. However, despite a rapid advancement and promising results of gene therapies based on modified viral vectors [5] some concerns remain about their safety [6]. As a result, non-viral vectors hold promise as a less dangerous and potentially more effective alternative, and various vectors based on liposomes, polycations, metal nanoparticles, copolymers, etc., have been developed [7]. As a result, a transfection vector based on thiolated poly(ethylene glycol)-poly(l-lysine) block copolymer (PEG-PLL) was developed, which responded to the reductive condition mimicking the intracellular environment [8]. All these systems have specific advantages and disadvantages, but one of the main problems is that the efficiency of existing ways for non-viral DNA delivery is still low. Hence, the search for new non-toxic and potent non-viral candidates capable of DNA delivery into mammalian cells continues, while clay nanoparticles are considered as candidates for DNA delivery.

Indeed, clay minerals such as montmorillonite, illite and kaolinite have been proposed as perspective DNA delivery vectors due to their excellent ability to bind and protect DNA from degradation [9] and even to transform microorganism cells in soil [10,11]. Additionally, clay minerals are generally non-toxic and have been widely administered by humans for medical purposes since ancient times as cures for different gastrointestinal disorders, such as diarrhea, or for poisoning prevention [12]. Among other clay minerals, montmorillonite is arguably the most thoroughly studied in regard of nucleic acid binding [13]. It turned out to be an effective protector for orally administered DNA from stomach acidic environments and DNA-degrading enzymes, enabling a successful plasmid DNA delivery into mural small intestine cells [14]. DNA-binding properties of halloysite are not studied well so far, if compared with other clays, although halloysite nanotubes have already been reported as a promising drug and cosmetic carriers [4,15,16,17] and successfully used for tissue engineering applications [18]. Halloysite nanotubes constitute a clay mineral with unique properties because of the oppositely charged outer and inner surfaces both of which can be used for binding various macromolecules. The reported cation exchange capacity of halloysite ranges from 0.1 to 0.7 mol/kg [19]. The halloysite nanotubes can be further modified to change their charge, colloid stability, lumen size [16] or rendering them stimuli-responsive [20]. Halloysite nanotubes have also been used as nanocontainers for delivery of antimicrobial dyes [21], paclitaxel [22], curcumin [23,24], antibiotics [25] and cell labeling agents [26]. Recently, the use of halloysite nanotubes-carbon dots hybrids as non-viral vectors for oral gene delivery was proposed [27].

Some attempts were made to tailor halloysite with DNA using high speed vibration milling process [28,29], which is a rather destructive approach hardly compatible with targeted gene delivery technique, or via grafting the nanotubes with polyethyleneimine (PEI) [30], γ-aminopropyltriethoxysilane (APTES) [31] or polyamidoamine [32]. Unfortunately, most of the polycations, including PEI, are cytotoxic [30], which stimulated us to develop a straightforward way for facile modification of halloysite clay nanotubes with DNA, avoiding the use of any potentially toxic additives. Here we report our results, which allow for efficient labelling of halloysite with nucleic acids, thus expanding the use of halloysite in biotechnology. We also show the self-assembly of DNA-halloysite hybrids on surfaces, which may find applications in hair surface engineering [17].

## 2. Results

**Figure 1:**
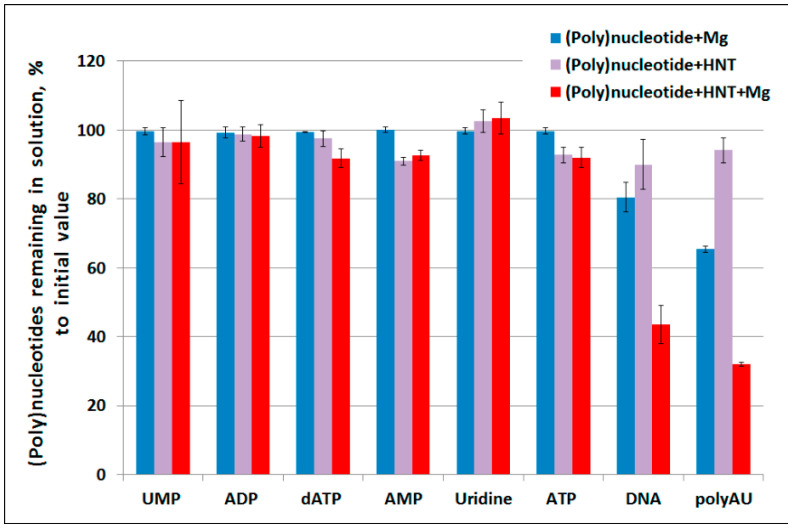
Binding of different (poly)nucleotides by halloysite nanotubes (HNT) with or without MgCl_2_ (10 mM).

To assess the binding of DNA to halloysite we used an approach similar to one described by Beall et al. [33], where the authors analyzed oligonucleotide binding properties of montmorillonite by measuring absorption at 260 nm. This method is often applied for measuring nucleic acid adsorption by clays [9,34,35] but one cannot expect the same adsorption results because of different chemical structures of various clay types. Our study revealed that halloysite only slightly bound nucleotides (AMP and ATP demonstrated the best binding (up to 10% of initial nucleotide quantity)) and did not bind uridine (Figure 1). Some nucleotide species such as AMP and ATP were bound by halloysite both in the presence and absence of MgCl_2_. Larger molecules such as DNA and polyAU were significantly bound by halloysite only when MgCl_2_ was present. The presence of MgCl_2_ alone was sufficient to decrease DNA and polyAU content in the supernatant; however, addition of halloysite further increased DNA and polynucleotide sorption.

Measurements of ζ-potentials have shown that all compounds were bound by halloysite both in the presence and absence of MgCl_2_ because changes in halloysite ζ-potential after interaction with a nucleotide species were detected in every case compared to untreated halloysite (Table 1). The specimens used for ζ-potential measurements were represented by a highly diluted (0.015 mg/mL) suspension of a thoroughly washed pellet of halloysite-(poly)nucleotide complex (obtained in the presence or absence of MgCl_2_), and so the specimens used for measurements hardly contained any free ions or low molecular-weight molecules, because they were lost during washing. The most prominent changes were observed after AMP, ATP, dATP and polyAU binding, while only a minor change was observed after halloysite incubation with uridine.

Halloysite nanotubes were sonicatied before the adsorption experiments to obtain a dispersion of individual nanotubes (the measured hydrodynamic diameter was 418.1 ± 8.529 and the ζ-potential was −26.5 ± 0.321). After the adsorption experiment, only pipetting and vortexing was applied (without sonication) during pellet washings and measurements in order not to provoke the release of bound substances. Although the pellet could be completely resuspended in all cases (except in the specimens where full length chicken erythrocyte DNA was present) even pristine halloysite nanotubes were highly agglomerated after the adsorption experiment, which was evidenced by their very large hydrodynamic diameter (Table 1). However, despite the low nucleotide absorption (Figure 1), the nanotube modification with ATP and dATP resulted in obtaining stable dispersions containing particles with low hydrodynamic diameters, thus suggesting using these nucleotides as low-consumable dispersants for halloysite biomedical applications (e.g., in drug delivery).

**Figure 2:**
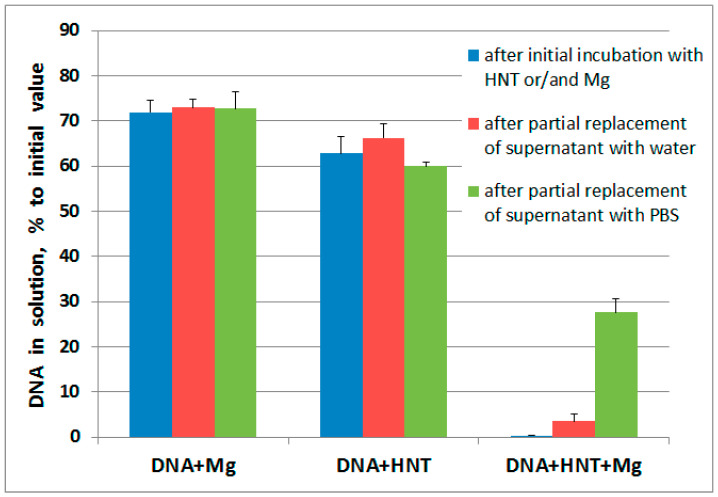
Ultrasonicated DNA content in solution after incubation with halloyste nanotubes or/and MgCl_2_ (100 mM) with the following partial replacement of supernatant with deionized water or PBS.

To increase the amount of DNA bound by the nanotubes we increased MgCl_2_ content in the incubation media up to 100 mM. Here, DNA that had undergone ultrasound fragmentation was used. Ultrasonicated DNA was completely bound by HNTs but only if MgCl_2_ was present (Figure 2).

Ideally, DNA delivered by a carrier vector must be detached from the delivering vehicle once the destination point is reached. Accordingly, the possibility of DNA release from halloysite nanotubes after an increase in pH to values observed in living organism under normal conditions was examined. In preliminary experiments we found that addition of the one forth (by volume) of PBS to the reaction mixture increased pH from 5.2 to 7.4. When one fourth of the supernatant volume was substituted with PBS, DNA was slightly released from HNTs while substitution with water was ineffective (Figure 2).

**Figure 3:**
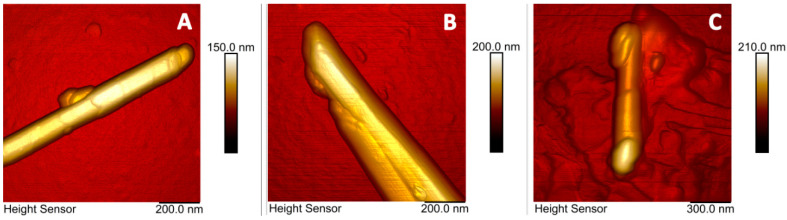
AFM images of pristine (**A**), Mg-(**B**) and Mg-DNA **(C)**-modified halloysite nanotubes.

Halloysite nanotubes were visualized using AFM (Figure 3), in addition, nanomechanical properties of pristine, Mg-, and Mg-DNA modified nanotubes were investigated. The atomic force microscopy method does not allow direct measurements of the quantity of DNA bound in the lumen vs. exterior surfaces of the HNT, but it allows to additionally support the presence of DNA on the clay surface and this was the reason of using this method in our work. While topographic features were not significantly different between the specimens, the covering of the nanotube surface with ultrasonicated DNA could be detected by measurements of adhesion force. Adhesion of Mg modified nanotubes was about three times higher than that of pristine nanotubes (HNT = 2.9 ± 1.5 nN; HNT + Mg = 10.5 ± 2 nN) indicating Mg adsorption on halloysite surface. Adsorption of DNA further increased the adhesion of nanotube surface (HNT + Mg + DNA = 17.5 ± 4.3 nN). There were no changes in the elasticity of halloysite surface.

Ultrasonicated DNA binding to halloysite was also confirmed by changes in halloysite ζ-potential (Table 1) and thermogravimetric data (Table 2). Based on the TGA data analysis, dATPNa was not loaded into the nanotubes (neither in the presence nor in the absence of MgCl_2_). Additionally, the degradation steps of MgCl_2_ were not detected in the hybrid systems (HNT-DNA-MgCl_2_ and HNT-dATPNa-MgCl_2_). Therefore, the amounts of MgCl_2_ in the loaded nanotubes were negligible.

**Figure 4:**
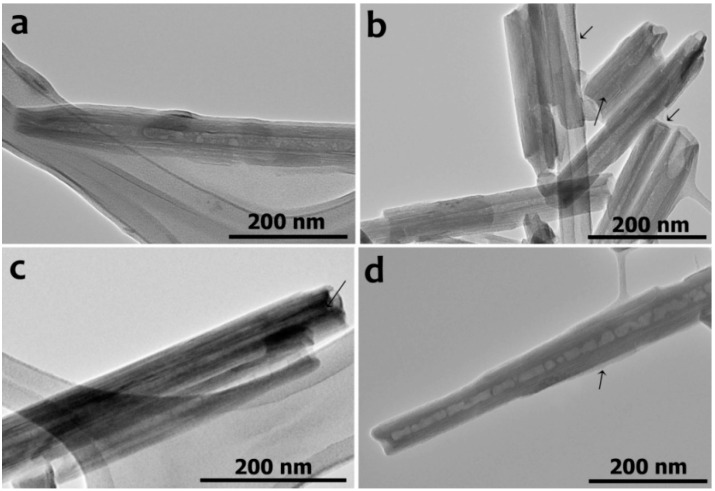
TEM images of halloysite nanotubes incubated with MgCl_2_ (**a**), ATP-Mg (**b**), AMP and MgCl_2_ (**c**), ultrasonicated DNA and MgCl_2_ (**d**). (The presence of organic matter is indicated by arrows).

The results of the HNTs interaction with some of the investigated species were visualized using TEM. We found that while nanotubes incubated with MgCl_2_ appeared to be hollow and clear on the outside, the tubes modified with AMP or ATP contained organic material within the lumen, and the surface of the tubes incubated with DNA in the presence of MgCl_2_ had a continuous coating on it (Figure 4). The results obtained resemble those of Khanna et al. [36] who visualized the binding of DNA to kaolinite and montmorillonite using TEM. Such techniques as X-ray diffraction (XRD), energy-dispersive X-ray spectroscopy (EDS) and scanning transmission electron microscopy (STEM) could be used to further corroborate the presence of organic matter in the clay structure, as it was demonstrated for characterization of the chitosan-montmorillonite nanocomposites [37].

**Figure 5:**
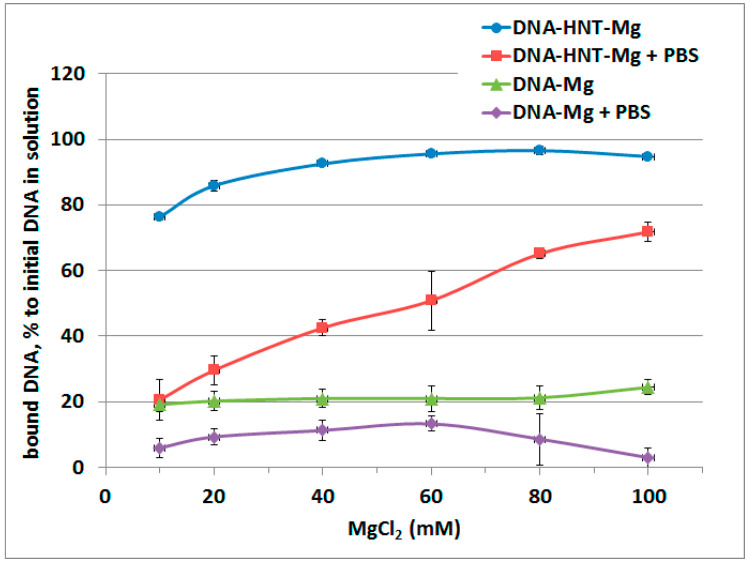
The amount of DNA bound to halloysite after initial incubation with different concentrations of MgCl_2_ followed by incubation in PBS solution.

To find out the MgCl_2_ concentration that would be optimal for DNA binding and release by the nanotubes a set of MgCl_2_ concentrations was tested and the optimal concentration was found to be between 40 and 60 mM (Figure 5). There was no further substantial increase in DNA binding to halloysite after about 50 mM MgCl_2_ concentration.

**Figure 6:**
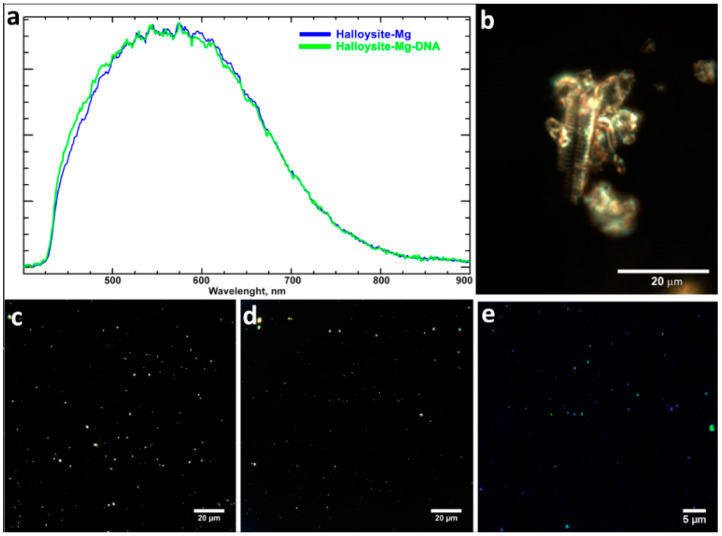
Averaged spectral profiles collected from halloysite-Mg (blue) and halloysite-Mg-DNA (green) particles (**a**), dark-field images of partially dissolved DNA (**b**), halloysite-Mg (**c**), halloysite-Mg-usDNA (**d**) particles and hyperspectral mapping of halloysite-Mg-DNA particles (**e**).

To further characterize halloysite nanotubes modified with usDNA an enhanced dark-field hyperspectral microscopy was performed (Figure 6). Figure 6a demonstrates an averaged spectrum for halloyste-Mg and halloysite-Mg-DNA with a peak at 550 nm. After nanotube modification with usDNA in the presence of MgCl_2_ a widening of the spectra was observed but the band located around 550 nm was retained. Reference spectral library collected from a DNA specimen was applied to detect DNA on HNTs particles using the ENVI spectral angle mapper function (threshold = 0.3 radians). Green pixels in Figure 6e mark the HNTs particles bearing DNA. In the field of view of a hyperspectral image 27% particles were mapped as HNT-Mg-DNA, while 73% were not mapped. Transverse striations visible on soaked and partly dissolved DNA fibers in a dark-field image of a DNA specimen (Figure 6b) were observed previously in the works of Livolant and coworkers and were explained as supramolecular ordering of DNA into liquid chrystalline structures (for example, see [38]).

**Figure 7:**
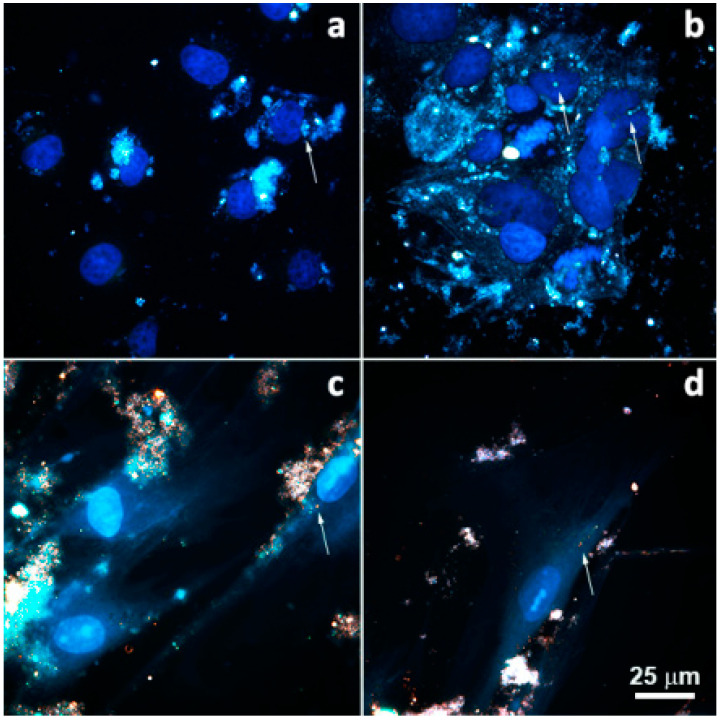
Dark-field images merged with fluorescence images of malignant (A549 (**a**), Caco-2 (**b**)) and non-malignant (HSF (**c**), MSC (**d**)) cells after 24 h incubation with halloysite nanotubes (nanotubes are indicated by arrows).

Assessing vehicle cell internalization is a necessary step in studies devoted to creation of novel transfection vectors. Pristine halloysite nanotubes were internalized by both malignant (Caco-2, A549) and non-malignant (MSC, HSF) cells (Figure 7), substantiating their use as vehicles for drug and nucleic acid delivery.

**Figure 8:**
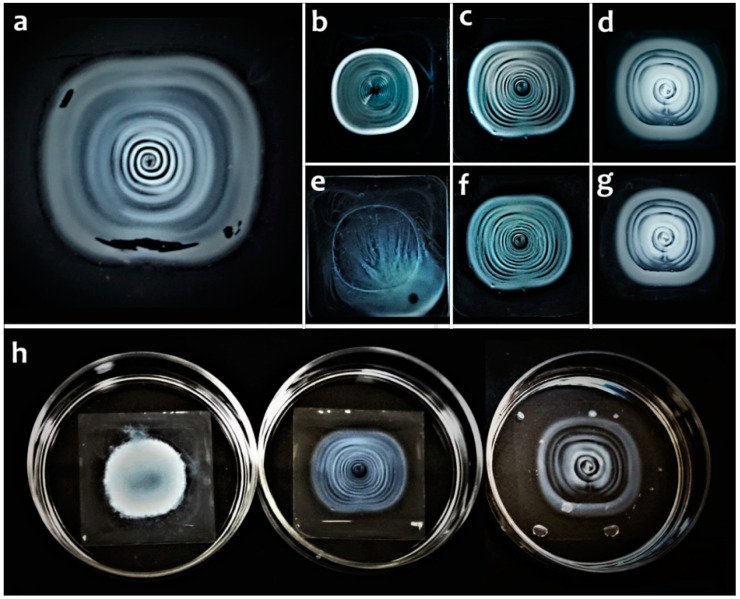
Concentric structures made from 1% suspensions (*w*/*v*) of halloysite-Mg-DNA (**a**,**d**,**g**), halloysite-PSS (**b**,**e**), halloysite-PSS-Mg-DNA (**c**,**f**) before (**a**–**d**) and after immersion in water (**h**) and drying (**e**–**g**).

Halloysite nanotubes are a perspective material for obtaining complex nano-featured coatings through evaporation-induced nanotube self-assembly process [39,40,41]. Previously, pristine nanotubes [42] or nanotubes stabilized by anionic polyelectrolyte PSS [43,44] were used for making concentric ring structures. In this work, the possibility of using DNA modified halloysite nanotubes for complex ring pattern formation was tested. Controlled solvent evaporation from a drop of 1% (*w*/*v*) suspension of halloysite nanotubes modified by ultrasonicated DNA in the presence of MgCl_2_ resulted in the appearance of spiral-like pattern (Figure 8a,d). The obtained patterns were very robust, e.g., a pattern made from 1% halloysite-PSS suspension immediately broke down after water addition while patterns made from 1% halloysite-Mg-DNA or 1% halloysite-PSS-Mg-DNA suspensions were unaffected by water and fully retained their appearance (Figure 8).

## 3. Discussion

An extensive literature exist about the binding of RNA, DNA and their components by clays [9,13,36,45], and a possible role of clays as catalysts in prebiotic RNA synthesis and the origin of life was hypothesized [46]. There were also successful attempts to amplify clay bound DNA by polymerase chain reaction [47,48] that have demonstrated that the amplification process could even proceed without DNA release from the clays [48]. Furthermore, the differences in structure and charges between clay minerals were reported to affect the DNA amplification because of varying tightness of DNA binding on different clays [48]. Halloysite belongs to clay minerals of kaolin group and occurs mainly as nanotubes of 700–1000 nm in length, with the outer diameter of 40–60 nm and the inner diameter of 10–15 nm; although shorter and longer nanotubes are also commercially available [16]. It is a hydrated aluminium silicate comprised of tetrahedral and octahedral sheets (1:1 phyllosilicate) and the tubes are formed as a result of layer rolling caused by the dimensional misfit between the octahedral and tetrahedral sheets in halloysite [49]. Halloysite surface charge is negative and rather stable at different pH, while the interior surface is positively charged [19,50]. Halloysie isoelectric point (pI) was found to be 3.8 [51], while the pI of DNA is about 5, and that of RNA is 2–2.5 [13]. Thus, all interacting components are negatively charged at pH above 5 used in our binding experiments, and direct electrostatic interaction between them is impossible. That is why Mg^2+^ cations were applied to form cation bridges and facilitate (poly)nucleotide binding to halloysite, as it was proposed that cations can form bridges between the phosphate groups of DNA and the negatively charged silica layer on the clay surface [33]. The observed somewhat higher sorption of substances containing several phosphate groups such as ATP, dATP and polynucleotides and no sorption of uridine which does not bear any phosphates supports this mechanism of binding. A complex of methods can be used to further explore the binding mechanism and cation bridge formation, such as specular neutron reflection (NR) and attenuated total internal reflection infrared spectroscopy (ATRIR) [52] or a combination of X-ray diffraction (XRD), inductively coupled plasma optical emission spectrometry (ICP-OES), X-ray photoelectron spectroscopy (XPS), and Fourier transform infrared spectroscopy (FTIR) [53].

However, a possibility that small negatively charged molecules can also penetrate inside positively charged halloysite nanotube lumen cannot be ruled out, because such species as AMP were bound by halloysite with equal effectiveness both in the absence and presence of Mg^2+^, indicating that the bulk of AMP molecules was adsorbed in the HNT lumen. The equal binding of ATP by halloysite nanotubes regardless of MgCl_2_ presence in the reaction medium was most probably related with the fact ATP was already represented by magnesium salt of ATP and additional Mg^2+^ was not needed for binding. According to UV absorbance measurements of nucleotides remaining in the supernatant after the adsorption experiment, ADP did not bind completely, while dATP was bound only when Mg^2+^ was present, most likely because these molecules were represented by ADP Na_3_ and dATP Na salts and Na could hinder the interaction. The slight difference between ADP and dATP binding in the presence of MgCl_2_ suggests that at least three phosphate groups are required for appreciable cooperative binding of nucleotide phosphates with halloysite. Since Mg was required for perceptible dATP binding by the nanotubes one can suggest that tri-phosphates are bound on the outer nanotube surface through Mg bridges, but the possibility of nucleotide di- and tri-phosphates binding both in the lumen as well as on the outside of the nanotubes could not be completely ruled out.

Despite the fact that 260 nm absorbance measurement did not demonstrate binding of ADP or dATP by halloysite, ζ-potential measurements demonstrated that all studied substances were bound by halloysite although the degree of binding remained uncertain. The proposed preferential binding of AMP inside the lumen and DNA on the outer surface of the tubes was confirmed by TEM images (Figure 4). Unfortunately, the location of ATP binding could not be unambiguously visualized during TEM investigations. It was hypothesized earlier that DNA could be bound in the lumen of the nanotubes [16]; however, as a polyphosphate molecule DNA could be bound also at the edges of the nanotubes, considering the well documented phosphate adsorption by the reactive sites exposed at the edges of kaolin mineral particles [49]. Based on SEM and TEM investigations, Khanna et al. [36] also made a similar conclusion that the binding of *Bacillus subtilis* chromosomal DNA occurred primarily on the edges of montmorillonite and kaolinite clays, although some binding was also visualized on the planar surfaces of the clays. Moreover, X-ray diffraction analysis demonstrated that the basal spacing of montmorillonite and kaolinite were not changed by DNA binding indicating that DNA did not significantly intercalate the clays [36]. In our work, the binding of DNA on the outer surface of the nanotubes was also confirmed by a difference in adhesion forces between pristine and DNA covered halloysite nanotubes measured using AFM. To further characterize the structure of the obtained clay/DNA complexes the small angle neutron scattering technique (SANS) is very useful, which was previously applied for studying the structure of anionic surfactant functionalized halloysite nanotubes [54] and halloysite/bioploymer hybrids [55].

Because direct injection of bare DNA for purposes of gene therapy is only applicable to some tissues such as muscles [56], the use of a viral or non-viral vector is required in most cases. The efficiency of direct gene transfer is low and variable [57]. Even if DNA is successfully taken up by cells only a very small proportion of it enters the nucleus as it was reported by Loyter et al. [58], where about 1–5% mammalian cells contained DNA in the nucleus after exogenous addition of DNA-calcium phosphate complex [58]. Cationic polymers have been long considered as perspective carriers for nucleic acid delivery, and efforts are now mostly applied to making them responsive for such stimuli as acidic environment to allow them to release their cargo in the endosomes, followed by endosomal membrane rupture and the cargo escape to cytoplasm [59]. Some nanoparticle mediated transfection methods have been developed, for example those based on Au nanoparticles [60].

The main challenge for successful gene delivery using any kind of a vector system is to find a balance between efficient DNA binding and protection by a vector and timely DNA cargo release after reaching the destination point [61]. That is why the development of a specific vector system for each tissue type is most probably required [61]. For example, a very high protection of DNA is needed in case of oral delivery. Clay minerals are especially promising artificial gene delivery vectors for oral administration because of their widely recognized non-toxicity (which must be individually tested for every new material, though) and many centuries-long history of medical oral application as anti-diarrhea and anti-poisoning preparation both in human and animals [62]. A successful oral delivery of plasmid DNA using montmorillonite was already demonstrated in mice [14].

But, as it was already mentioned, a good transfection vector must not only be capable of binding DNA molecules effectively, it should also easily release its cargo when the appropriate circumstances of the cell interior are reached. To assess the ability of halloysite to release DNA after entering the human organism PBS was added to the reaction mixture to coarsely model the intra-organism environment by elevating the mixture pH to 7.4. DNA release was observed after addition of PBS solution, and two mechanisms can be credited with enhancing DNA release: the first is related with pH increase and the second involves competition of PBS components, for example, phosphate anions, with DNA for halloysite binding sites. It was shown for different clay minerals that the amount of absorbed DNA, RNA and nucleic acid bases significantly decreases with increasing pH [13]. Nucleic acid bases can be adsorbed by clay species [45,63], especially at low pH, where protonation and hence positive charges of nucleic acid bases contribute to their binding to negatively charged clays [63]. However, a comparatively high kaolinite isoelectric point results in no detectable binding of nucleic acid bases (except for adenine) by kaolinite even at pH as low as 2 [63]. We suggest that DNA was released because of competition with phosphate anions rather than increase in pH, because both DNA and halloysite surface are negatively charged at pH 7.4 and the stability of Mg^2+^ bridges is not disturbed under these conditions. Thus, the transition from pH 5 to pH 7.4 probably contributed only insignificantly to the DNA desorption process in our work and the main mechanism was related to competition from anions in PBS. The competitive displacement with anionic biomacromolecules is regarded as a mechanism potentially useful for DNA cargo release inside the target organism [59]. The surface of halloysite nanotubes could be additionally modified by polyelectrolytes using layer-by-layer approach to protect the nucleic acid cargo from competitive displacement by lipid phosphates encountered in cell membranes. However, even unprotected DNA bound to the sepiolite surface through cation bridges successfully crossed the cell membrane and was expressed in mammalian cells [64]. The binding and release of DNA by halloysite nanotubes shown here opens up a perspective of their similar application as vectors for oral DNA delivery.

Further studies on the influence of other cations on DNA interaction with halloysite nanotubes are needed to find the most efficient mediator of formation of nucleic acid-halloysite complexes. From the studies on other silicates (including nanoclays) [33,65] it could be expected that polyvalent cations will promote nucleic acid binding to halloysite nanotubes due to formation of cation bridges while monovalent cations will decrease the adsorption of polyphosphates due to screening the interaction between the clay silanol groups and the negatively charged phosphate groups of DNA. A direct correlation between the efficiency of DNA adsorption on sepiolite and the cation valence was found when using mono-, di,-tri-, and tetravalent cations, but too strong binding by polyvalent cations hindered the subsequent DNA release compared to divalent cations and decreased the transfection efficiency [64].

If halloysite nanotubes are to be used as DNA delivery vehicles, a method is desirable to track DNA modified halloysite nanotubes in cells and living tissues. Dark-field hyperspectral microscopy appeared as a useful tool for visualization and identification of nanoparticles in biological samples, allowing studies of non-stained and even non-fixed objects [66,67]. The spectral profiles obtained from halloysite-Mg and halloysite-Mg-usDNA were similar, but the halloysite-Mg spectrum was slightly red-shifted compared to the halloysite-Mg-usDNA one, indicating higher agglomeration of the sample, additionally confirmed by the dark field images. Mapping of DNA on clay nanotubes using hyperspectral microscopy was only partially successful, probably because of some differences in spectral profiles between the partly dissolved pure DNA used for obtaining a reference DNA library for mapping and usDNA bound to halloysite through Mg^2+^ ions.

In this work UV-spectroscopy was used for evaluation of (poly)nucleotide binding and release from halloysite nanotubes. A useful approach for studying release kinetics and cell penetration of a DNA-clay complex can be applied for further development of a halloysite based transfection vector, involving labelling DNA and polynucleotides with a fluorescent tag and using fluorescent microscopy and fluorometry for registering the results. A similar approach was applied by Castro-Smirnov et al. [64] to evaluate the efficiency of sepiolite nanoclay in short interfering RNA (siRNA) delivery into A673 cells by using FITC labelled siRNA.

Cell penetration of pristine and modified halloysite nanoclay was previously demonstrated in numerous studies [68,69,70,71]. In vitro studies showed that halloysite successfully delivered prodigiozin and brilliant green into cancer cells, which resulted in cancer cell death [21,70]. Moreover, a positive nucleus targeting of halloysie nanotubes could be achieved by covalent linkage of nitrogen doped carbon dots on the halloysite surface [27].

There are two main approaches for gene-based therapy, namely ex vivo and in vivo approaches, and in vivo applied gene therapy tools can be delivered orally, through the bloodstream or by direct intratumoral injection [72]. Nanoparticulate vehicles can be primarily used for targeting liver cells or solid tumors, because of discontinuous endothelia in these tissues, enabling the passing of particles of 100–200 nm in diameter [73]. Moreover, the combination of highly permeable endothelia and poor lymphatic drainage can result in increased accumulation of circulating nanoparticles in tumors [74]. However, halloysite nanotubes are non-biodegradable in blood and are not recommended for direct injections [75], suggesting preferential oral delivery for halloysite-based nucleic acid delivery systems. Additionally, the size of halloysite nanotubes may be an obstacle for delivery through blood vessels, although the size of the nanotubes depends on the nanotube origin [16] and methods were suggested to decrease the nanotube size [76].

The creation of a ready-to-use transfection vector based on halloysite nanotubes was not the purpose of this study, and further steps must be implemented before the nanoclay can be regarded as a carrier for therapeutic nucleic acids, including assessing nucleic acid intactness after release from the nanoclay, the binding and release efficiencies of oligonucleotides of varying lengths, nucleic acid release kinetics under modeled environments (blood plasma or cell culture media), and in vitro and in vivo transfection experiments. The adsorption of nucleic acids in/on clays strongly depends on the nucleic acid size and conformation [13]. Thus, testing of adsorption/desorption of polynucleotide molecules of specified lengths and conformations under varying conditions is needed to fully characterize the nucleic acid packaging capacity of halloysite nanotubes and further understand their suitability for being used as carriers for therapeutic nucleic acids. In a recent study on a potential application of another nanoclay, sepiolite, for DNA transfer into mammalian cells, the quality of desorbed plasmid DNA was assessed using electrophoresis, and it was found that DNA desorbed from the nanoclay fully retained its structure (super-coiled, linear, open circle) [64]. As halloysite nanotubes are suggested as vectors for oral nucleic acid delivery, testing of halloysite behaviour under simulated gastric environments is also required. In vivo studies demonstrated that oral delivery of an antimicrobial nanocontainer based on halloysite nanotubes loaded with curcumin and protected with a dextrin outer layer was effective in selective suppression of the overgrowth of pathogenic bacteria in *Caenorhabditis elegans* nematodes [24]. The release kinetics of the anticancer drug paclitaxel encapsulated in halloysite coated with the pH-responsive polymer poly(methacrylic acid-co-methyl methacrylate was evaluated in simulated gastric and intestinal conditions, and a triggered drug release pattern was observed [22].

The results described in this report may be useful also for development of DNA based nanomaterials for various applications considering some unique properties of DNA. For example, temperature-responsive nanocapsules were prepared by layer-by-layer technique where a DNA layer was included to benefit from DNA ability to undergo temperature dependent denaturation [77]. The combination of oligonucleotides with PNIPAM allowed formation of stable DNA-polymer thin films and micelles [78]. Wrapping of halloysite nanotubes in DNA was also suggested as a means of increasing halloysite water-dispersibily for using it as a carrier for drug delivery [29]. Based on the results obtained here, ATP Mg could be proposed as another agent stabilizing halloysite dispersions for biological and medical applications, because of the ubiquitous presence of ATP in living organisms and the small amount of ATP needed for halloysite treatment.

Different non-toxic systems based on natural polyelectrolytes such as DNA can be designed and additionally reinforced with natural clay minerals if needed. Halloysite is widely applied as a reinforcing agent for polymeric materials [79] while DNA finds numerous applications in photonics and optoelectronics as a glue or template for metal nanoparticle assembly into complex structures [80]. Evaporation of nanoparticle suspensions under controlled conditions was proposed as a relatively easy method for nanoparticle self-assembly into complex patterns [81,82,83,84]. The method is based on a so called coffee-ring effect, where an evaporating droplet of a nonvolatile solute suspension leaves a stain with a dense rim, because faster solvent evaporation from the drop edges leads to a local solute accumulation and “pinning” of the soild-liquid-vapour contact line [85]. When the three-phase contact line of the droplet is pinned, the liquid evaporating from the drop edges is replenished by the liquid from the drop interior with continuing solutes outflow toward drop borders. If a droplet evaporates under restricted environments, a complex regular pattern can be formed due to the controlled repeated pinning and depinning (“stick-slip” motion) of the three-phase contact line [86]. Various biomedical applications were proposed for nano-patterned surfaces obtained this way using halloysite nanotubes, such as capturing circulating cancer cells [39,40] or directed cell growth [42]. In a “curve-on-flat” restricted geometry used in this study, a drop of a particulate dispersion is placed in a narrow slit between a curved upper surface and a flat substrate, which allows liquid evaporation only at the edges of the dispersion meniscus held between the upper surface and the substrate by a capillary force. Evaporation leads to pinning of the contact line while the capillary force induces contact line depinning and its jump to a new position as soon as the critical value of the meniscus contact angle is reached. The repeated pinning and depinning of the contact line results in particle depositions in the form of concentric structures [86].

Safety is obligatory for biomedical materials, thus using non-toxic components such as clay nanotubes and DNA is advantageous for creating novel materials intended for being in contact with living matter. Low toxicity of halloysite nanotubes for prokaryotic and eukaryotic organisms was repeatedly shown [24,71,87,88,89,90]. Here, evaporation induced self-assembly of DNA-Mg-modified halloysite nanotubes yielded a complex pattern similar to those previously obtained from pristine [42] or PSS-modified nanotubes [43], however, the pattern obtained from DNA-Mg-modified halloysite was more spiral-like rather than simply concentric. The high durability of the obtained dense nano-patterned coating is beneficial for biomedical applications involving a long-term cell cultivation or liquid filtration along patterned surfaces.

## 4. Materials and Methods

### 4.1. Materials

Halloysite nanotubes were obtained from Applied Minerals Inc. (Brooklyn, NY, USA). Chicken erythrocyte DNA, polyadenylic–polyuridylic acid (polyAU), uridine-5′-monophosphate disodium salt (UMP Na_2_), adenosine 5′-diphposphate, trisodium salt (ADP Na_3_), 2′-deoxyadenosine 5′-triphosphate sodium salt (dATP Na), adenosine monophosphate (AMP), uridine (Reanal, Budapest, Hungary), adenosine 5′-triphosphate magnesium salt (ATP Mg), poly(styrene sulfonate) sodium salt (PSS) (average mol. weight ∼70,000) (Sigma-Aldrich, Darmstadt, Germany) were used as purchased, without further purification.

### 4.2. Methods

#### 4.2.1. Binding and Release of DNA, Nucleotide Phosphates or Uridine

The binding experiments were performed in 1 mL of distilled water (dH_2_O) (pH 5.2–5.4). The reaction mixture included a nucleotide species (DNA, a nucleotide phosphate or uridine), halloysite (1.5 mg/mL) and optionally MgCl_2_ (10 mM). Nucleotide phosphate or uridine content was 0.15 µM (which corresponded to the weight ratio of halloysite to nucleotide about 20–40:1). DNA or polyAU content was 50 µg/mL, corresponding to the halloysite:(poly)nucleotide weight ratio of 30:1. In control samples either halloysite, MgCl_2_ or both were omitted. The mixture was incubated for 1.5 h at room temperature, centrifuged at 3280× *g* (Eppendorf Minispin Plus, Hamburg, Germany) and an aliquot (250 µL) was taken, diluted 5 times and the absorbance was measured at 260 nm using a Lambda 25 spectrometer (Perkin Elmer, Waltham, MA, USA). The pellet was washed 3 times with distilled water, resuspended, and its ζ-potential and hydrodynamic size was measured using a Zetasizer Nano ZS instrument (Malvern Instruments, Malvern, UK) and standard Malvern disposable capillary cells at 25 °C in deionized water [91,92,93]. Another approach is usually applied in clay mineralogy to obtain clay-Mg-nucleic acid complexes. At first stage, the clay surface is saturated with Mg^2+^ by incubation with excess of MgCl_2_ and rinsed until all of the Cl^−^ is removed [94]. Although this procedure is advantageous because it allows calculation of the amount of Mg^2+^ bound to the clay surface, our approach consisting in incubation of clay with nucleic acids in the presence of Mg is more straightforward and usually applied for preparing clay-nucleic acid complexes for the purposes of gene delivery to cells [64].

#### 4.2.2. Binding and Release of Fragmented DNA

DNA stock solution in distilled water (1–2 mg/mL) was prepared and subjected to ultrasound fragmentation at 50% amplitude using Bandelin SonoPlus sonifier (Bandelin, Berlin, Germany). The reaction mixture included (in the final concentrations) fragmented DNA (50 µg/mL), halloysite or (1.5 mg/mL) and MgCl_2_ (100 mM). The ratio of DNA (nucleotide) to halloysite was 1:30. Mixtures containing DNA, MgCl_2_, and halloysite with MgCl_2_ were used as controls. After 1.5 h incubation at room temperature the mixture was centrifuged for 5 min at 600× *g* (Heraeus Pico 17, Thermo Fisher Scientific, Waltham, MA, USA), an aliquot (250 µL) was taken from the supernatant, diluted fivefold and absorbance was measured at 230, 260, 280 nm to assess the binding of DNA to halloysite. This centrifuge speed was chosen empirically, as it was the speed that allowed settling down all clay particles without formation of a too dense clay pellet, because the pellet was to be resuspended at the next stage after addition of PBS.

The equal amount (250 µL) of dH_2_O or phosphate buffered saline (PBS) was added to the reaction mixture, vortexed and incubated overnight at room temperature to estimate DNA release. The preliminary experiments showed that addition of such amount of PBS was enough to shift the mixture pH from 5.2 to 7.4. Next day the mixture was centrifuged at 600× *g*, an aliquot (250 µL) was taken, diluted 5 times and its absorbance was measured at 260 nm. To find out the MgCl_2_ concentration that would be optimal for ultrasonicated DNA (usDNA) binding and release by the nanotubes the set of MgCl_2_ concentrations in the range of 10 to 100 mM was tested in a system containing usDNA (25 µg) and halloysite (750 µg) in 0.5 mL. After 1.5 h incubation, the solution was centrifuged 5 min at 600× *g* (Heraeus Pico 17, Thermo Fisher Scientific) and a half of the supernatant (250 µL) was replaced with PBS. The removed aliquot of supernatant was used for measuring usDNA content by A260 absorbance. After re-suspending the sediment the system was incubated overnight at room temperature, centrifuged and an aliquot of supernatant (250 µL) was used for measuring usDNA content. The amount of us DNA bound under different conditions was calculated based on the data obtained. All experiments were conducted in triplicate and means with standard deviations are presented in tables and figures.

#### 4.2.3. Thermogravimetric Analysis

The amount of ultrasonicated DNA and selected nucleotide species associated with halloysite nanotubes was assessed using a thermogravimetric analyzer Q5000 IR (TA Instruments, Vimodrone, Italy). The measurements were performed by heating the specimens in the temperature range of 25–700 °C at 10 °C/min rate in the atmosphere of nitrogen. The weight percentage of components was calculated as described by Lisuzzo et al. [95].

#### 4.2.4. Microscopy

The surface of pristine and modified halloysite nanotubes was visualized using a Dimension Icon atomic force microscope (Bruker, Billerica, MA, USA) operating in PeakForce Tapping mode. AFM imaging was performed in air using ScanAsyst-Air probes (Bruker) (nominal length 115 µm, tip radius 2 nm, spring constant 0.4 N/m). The images were obtained at 512–1024 lines/scan at 0.8–0.9 Hz scan rate. The raw AFM data was processed using Nanoscope Analysis v.1.7. software (Bruker). Adhesion was calculated from particle surface areas sized 100 × 100 nm.

Additionally, the images of halloysite nanotubes after interaction with MgCl_2_, ultrasonicated DNA, AMP and ATP-Mg were obtained using Hitachi HT7700 Exalens (Hitachi, Tokyo, Japan) transmission electron microscope (TEM) operating at 100 kV in TEM mode. Diluted halloysite nanotube suspension was drop-cast onto formvar-coated copper grids (Agar Scientific, Stansted, UK) and left to evaporate, then the nanotubes were imaged at 120 V accelerating voltage.

Enhanced dark-field and hyperspectral microscopy was performed using an Olympus BX51 microscope (Tokyo, Japan) equipped with CytoViva^®^ high-aperture dark-field condenser (CytoViva, Auburn, Ala., USA). The dark-field images were obtained using Exponent (Stable Microsystems, Godalming, UK) software (Version 7.0). Hyperspectral data were recorded at 2 nm spectral resolution in the 400–1000 nm range. Recording and mapping of hyperspectral data were performed using ENVI (Harris Geospatial Solutions, Broomfield, CO, USA) software (Version 4.8). To obtain a DNA specimen for hyperspectral imaging a piece of dry DNA was placed on a glass slide and dissolved by gradual addition of dH_2_O to enable precise localization and focusing on DNA in the specimen.

#### 4.2.5. Halloysite Internalization by Cells

Adenocarcinomic human alveolar basal epithelial cells (A549), colorectal adenocarcinoma cells (CaCo-2), rat adipose-derived mesenchymal stem cells (MSC) and human skin fibroblasts (HSF) were cultured in α-MEM (Sigma-Aldrich, Darmstadt, Germany) supplemented with 10% of fetal bovine serum (Thermo Fisher Scientific), 100 IU/mL penicillin, 100 μg/mL streptomycin and 2 mM L-glutamine in humidified atmosphere with 5% CO_2_ at 37 °C. Cells (1 × 10^5^ per well) were seeded on round coverslips in 6 well cell culture plates (Corning, Corning, NY, USA) and cultured for 24 h in CO_2_ incubator at 37 °C. Halloysite nanotubes in PBS were added to each well to final concentration 100 μg per mL and cells were incubated for 24 h. Then, the culture medium was aspirated, cell were washed with PBS and fixed with 4% paraformaldehyde solution in PBS. After cell washing with PBS the nuclei of the cells were stained with 4′,6-diamidino-2-phenylindole (DAPI) according to the standard protocol.

Dark-field images were obtained using an Olympus BX51 (Olympus, Tokyo, Japan) upright microscope equipped with a CytoViva^®^ enhanced dark-field condenser with a halogen light source (150 W) Fibre-Lite DC-950 (Dolan-Jener, Boxborough, MA, USA) and control module ProScan III (JH Technologies, Fremont, Calif., USA). Images were obtained using acquisition software for visualization Exponent (Stable Microsystems, Godalming, UK) (Version 7.0). An X-cite 120Q wide-field fluorescence microscope excitation light source (Excelitas Technologies, Waltham, MA, USA) and CytoViva^®^ Dual Mode Fluorescence system equipped with the Triple Pass Filter were used to image DAPI nuclear staining with transmitted fluorescence illumination imaging, exposure time was 100 μs. The dark-field images were merged with transmission fluorescence images using the freely available image processing GIMP.

#### 4.2.6. Fabrication of Concentric Rings from DNA-Modified Halloysite Nanotubes on a Solid Substrate

An aliquot (400 µL) of 1% (*w*/*v*) water suspension of halloysite nanotubes modified with Mg and ultrasonicated DNA (weight ratio of MgCl_2_:DNA = 1:10) was placed within a narrow gap between a square glass pyramid (16 × 16 mm) and a degreased cover-glass. The pyramid was fixed firmly above the cover glass so that the distance between the pyramid summit and a cover-glass was about 0.5 mm. The whole installation remained motionless during drying in an oven Binder ED 23 (Binder, Tuttlingen, Germany) at 60–65 °C for 2–2.5 h. After the drying the pyramid mould was removed from the cover-glass and a pattern obtained on the glass surface was washed twice with deionized water. Water resistance of the pattern was studied by incubating it in distilled water and compared to the resistance of the patterns obtained from suspensions of halloysite modified PSS. To make a suspension of halloysite-PSS 1 g of PSS was dispersed in 50 mL distilled water and 1 g of HNTs was gradually added. The mixture was continuously stirred for 48 h at RT, centrifuged (20 min, 3500 rpm) and washed several times with distilled water. The resulting pellet was dried at 70 °C, ground, ultrasonically crushed and used for preparation of 1% (*w*/*v*) halloysite-PSS and halloysite-PSS-Mg-DNA suspensions. To prepare halloysite-PSS-Mg-DNA suspension halloysite-PSS was incubated with MgCl_2_ and usDNA (weight ratio MgCl_2_:DNA = 1:10) for 1.5 h at RT, centrifuged at 600× *g* for 5 min and washed 3 times with dH_2_O.

## 5. Conclusions

In this study, an easy way of halloysite nanotube modification with DNA was proposed. Compared to previous reports, where DNA was combined with halloysite through high speed vibration milling process, the binding of DNA to halloysite in solution in the presence of MgCl_2_ is a less destructive and more controllable way for obtaining DNA modified halloysite nanotubes. Moreover, the bound DNA could be partially released from the nanotubes under appropriate conditions, the percentage of released DNA depending on the amount of MgCl_2_ present during the binding process. Thus, modification of halloysite nanotubes with DNA can be potentially used for transferring information encoded in nucleic acids for the purposes of gene therapy. On the other hand, the high efficiency of DNA binding by halloysite may be beneficial for various technological applications, where DNA molecules serve as a natural anionic polymer, complementing the non-toxic natural clay nanotubes in novel composite materials or fastening nanoarchitectures built from clay nanoparticles. Thus, the results of this study add to our knowledge of remarkable ability of clay minerals to interact with nucleic acids, which can find practical application in designing different entities for technical or medicinal purposes.

## Figures and Tables

**Table 1:** ζ-potentials and hydrodynamic diameters of pure and (poly)nucleotide species treated halloysite in the presence or absence of MgCl_2_ (10 mM). In the case of ultrasonicated DNA binding was performed at MgCl_2_ (100 mM).

Substance	(Poly)nucleotide ζ-Potential, mV	HNTs with a Substance Bound		HNTs with a Substance Bound in the Presence of MgCl_2_	
		ζ-Potential, mV	Size, nm	ζ-Potential, mV	Size, nm
Pure halloysite nanotubes (HNTs)		−25.2 ± 0.2	2283 ± 53.84	−18.9 ± 0.3	4839 ± 20.22
UMP Na_2_	−20.3 ± 0.9	−29.9 ± 0.6	2242 ± 95.77	−23.5 ± 0.4	1251 ± 46.87
ADP Na_3_	−21.2 ± 1.3	−34.4 ± 1.1	601.4 ± 18.73	−20.6 ± 0.15	897.0 ± 39.83
dATP Na	−19.6 ± 1.8	−44.6 ± 0.11	557.2 ± 10.53	−26.2 ± 0.4	512.2 ± 4.5
AMP	−11.9 ± 0.8	−37.7 ± 0.6	1296 ± 48.57	−26.4 ± 0.6	958.6 ± 38.39
Uridine	−21.0 ± 1.2	−28.4 ± 0.3	5351 ± 256,0	−21.3 ± 0.15	2026 ± 72.9
polyAU	−39.7 ± 0.9	−40.2 ± 0.4	803.0 ± 35.66	−27.7 ± 0.4	644.8 ± 12.2
ATP Mg	−10.6 ± 4	−37.3 ± 0.4	561.7 ± 8.843	−31.0 ± 0.6	468.6 ± 5.546
Chicken erythrocyte DNA	−73.6 ± 2	-	-	-	-
Ultrasonicated DNA	−56,4 ± 11,6	−34,4 ± 1,1	708.2 ± 11.47	−30,8 ± 0,61	936.6 ± 25.32

**Table 2:** (Poly)nucleotide content in halloysite nanotubes according to thermogravimetric analysis.

Sample	Amount of (Poly)nucleotide Loaded to Halloysite, wt%
HNT-usDNA	0
HNT-usDNA+MgCl_2_	11.1
HNT-dATPNa	0
HNT-dATPNa+MgCl_2_	0
HNT-ATPMg	8.0
HNT-polyAU	1.4
